# Comparing Stress, Anxiety, and Depression in Adolescent Practitioners of Hatha Yoga and Recreational Sports: A Cross-Sectional Study

**DOI:** 10.7759/cureus.95723

**Published:** 2025-10-30

**Authors:** Vishnu Priya Puttraju, Jayanthi Srikanth

**Affiliations:** 1 Internal Medicine, King's College Hospital, London, GBR; 2 Community Medicine, Kempegowda Institute of Medical Sciences, Bangalore, IND

**Keywords:** anxiety, depression, sports, stress, yoga

## Abstract

Background: Adolescents are vulnerable to psychiatric conditions, with depression, stress, and anxiety being particularly common. Prior research suggests that physical activities such as recreational sports and yoga may benefit mental health. However, limited comparative data exist on these interventions for managing adolescent mental health.

Aim: This study aimed to examine whether stress, anxiety, and depression levels differ between adolescent yoga and sports practitioners.

Methods: A cross-sectional study of 190 adolescents was conducted, including 95 Hatha yoga practitioners and 95 recreational sports participants. Mental health outcomes were assessed using the Depression Anxiety Stress Scales (DASS-21). Independent samples t-tests and chi-square tests were used to compare groups, with p≤0.05 considered statistically significant.

Results: Among 190 adolescents (95 per group), recreational sports participants demonstrated significantly lower stress scores (11.75±7.44 versus 13.70±6.18) and better overall mental health outcomes compared with Hatha yoga practitioners. Notable confounding factors included substantial differences in practice duration and age distribution between groups.

Conclusion: This cross-sectional study found better overall mental health outcomes and lower stress scores among recreational sports participants compared to yoga practitioners. However, substantial confounding factors, including differences in practice duration and demographics, limit causal interpretation. These preliminary findings require validation through controlled studies before clinical recommendations can be made. The results suggest that school-based recreational sports programs may offer a cost-effective, scalable approach to adolescent stress management and primary prevention of mental health conditions.

## Introduction

Adolescence represents the transition from childhood to adulthood, a critical developmental period characterized by biological, psychological, and social changes that shape future health and well-being [1]. According to the World Health Organization (WHO), 14% of 10-19-year-olds experience mental health conditions, yet the majority remain undetected and untreated [2]. It was also estimated that almost half of these conditions begin by the age of 14 years [3]. The ability of adolescents to successfully navigate this stage has significant implications not only for their individual futures but also for the social and economic growth of a nation [4].

Lifestyle modifications, particularly physical activity, have been shown to reduce symptoms of anxiety and depression. Evidence suggests that both exercise and yoga have superior effects on mental health compared to inactivity, and their outcomes are comparable to established treatments such as cognitive-behavioral therapy and pharmacological interventions (e.g., sertraline and imipramine) [5,6]. High-energy or frequent aerobic exercise (three to five sessions per week) has been associated with reduced symptoms of depression and anxiety, along with improvements in self-esteem, body image, and overall mental health [6-8].

Recreational sports are defined as activities where the primary purpose is participation, with related goals of improved physical fitness, fun, and social involvement often prominent, and are usually perceived as being less stressful, both physically and mentally, with lower expectations regarding performance and commitment compared to competitive sports. Recreational sports encompass both aerobic and anaerobic activities that provide mental health benefits through cardiovascular stimulation and social interaction [9]. The social component inherent in many recreational sports may provide unique stress-buffering effects through peer support, sense of belonging, and collective efficacy [10].

Hatha yoga, in contrast, is a mind-body practice integrating postures (asana), meditation (dhyana), and controlled breathing (pranayama). This slow-paced style of yoga emphasizes relaxation and mindfulness, making it accessible even to beginners. We specifically selected Hatha yoga for comparison as it represents the most widely practiced form of yoga in educational and therapeutic settings, particularly among adolescents and beginners. Hatha yoga practice is characterized by low intensity, with metabolic rate generally in the range of 1-2 metabolic equivalents (MET), with average energy expenditure in asanas of 2.29 kcal/min [11-13]. Unlike more vigorous yoga styles such as Vinyasa or Ashtanga, which involve continuous flowing movements that can elevate heart rate comparable to aerobic exercise [14,15], Hatha yoga maintains a gentler pace with sustained posture holds and emphasis on breath awareness, representing a distinct mind-body intervention focused on parasympathetic nervous system activation rather than a cardiovascular training modality [12].

These two approaches represent fundamentally different physiological and psychological interventions: dynamic, social, metabolically demanding activities versus low-intensity, individual, introspective practices. This contrast makes them ideal for comparative study in understanding how different physical activity modalities may influence adolescent mental health.

Despite growing evidence supporting the role of exercise and yoga in managing depression and anxiety, relatively few studies have systematically compared their effects on stress in adolescents [16]. However, limited research comparing yoga and sports prevents us from predicting which activity would show better outcomes. Therefore, we employed an exploratory comparative approach to examine the distribution and prevalence of stress, anxiety, and depression symptoms between adolescents engaged in Hatha yoga versus recreational sports and to identify any differences in mental health patterns between these two physically active populations.

Given this critical developmental window when mental health conditions emerge, understanding how different physical activities influence adolescent mental health becomes essential for developing effective, accessible interventions that can be implemented during this vulnerable period [17]. Given that the majority of adolescent mental health conditions remain unrecognized and untreated, incorporating accessible practices such as yoga or recreational sports into school programs may represent a promising complementary approach [1].

## Materials and methods

Study design and setting

This cross-sectional study was conducted over a period of six months, from February to August 2019. Institutional Ethics Committee approval was obtained for the entire duration of the study (reference number: KIMS IEC/UG - 02/2019).

Participants and sampling

Participants were selected through purposive sampling. Inclusion criteria were adolescents aged 10-19 years who had been regularly practicing either recreational sports or Hatha yoga for at least six months, with a frequency of three to five sessions per week [18]. Exclusion criteria were individuals who practiced both yoga and sports, and those who were professional athletes.

Participant recruitment was conducted at private educational institutions and specialized activity centers in Bengaluru. Yoga participants were recruited from private schools offering yoga programs and dedicated yoga centers where Hatha yoga instruction was provided to adolescents. Sports participants were recruited from private sports coaching centers offering badminton, swimming, basketball, and football training programs. All participants attended private schools in urban Bengaluru, ensuring a relatively homogeneous educational and urban environment, although we acknowledge that socioeconomic variability within this population was not systematically assessed or controlled for in this study design.

Sample size

The study sample size was estimated using prevalence rates of depression, anxiety, and stress from a comparable study conducted among adolescents in Bengaluru [19]. Considering a margin of error of 10% and a confidence level of 95%, the minimum sample size was calculated to be 95 participants in each study arm.

Data collection tools and procedures

Data were obtained after securing informed written consent from parents/guardians and assent from participants. Confidentiality was assured, and each subject completed a structured questionnaire consisting of two parts: sociodemographic details, including age, gender, address, education, and the number of hours of sport/yoga practiced per week, and the Depression Anxiety Stress Scales (DASS-21), a validated screening tool for assessing depression, anxiety, and stress in adolescents [20-22].

The questionnaire comprises 21 items distributed equally across three subscales: depression (items 3, 5, 10, 13, 16, 17, and 21), anxiety (items 2, 4, 7, 9, 15, 19, and 20), and stress (items 1, 6, 8, 11, 12, 14, and 18). The depression subscale assesses symptoms such as dysphoria, hopelessness, devaluation of life, self-deprecation, lack of interest/involvement, anhedonia, and inertia. The anxiety subscale evaluates autonomic arousal, skeletal muscle effects, situational anxiety, and subjective experience of anxious affect. The stress subscale measures difficulty relaxing, nervous arousal, and being easily upset/agitated, irritable/over-reactive, and impatient. Participants rated how much each statement applied to them over the past week using a 4-point Likert scale: 0 = "did not apply to me at all," 1 = "applied to me to some degree, or some of the time," 2 = "applied to me to a considerable degree, or a good part of the time," and 3 = "applied to me very much, or most of the time." Scores for each subscale were calculated by summing the responses to the seven relevant items and multiplying the sum by two. This multiplication makes the DASS-21 scores comparable to those from the original 42-item DASS. The resulting scores for each subscale were then classified into severity categories (normal, mild, moderate, severe, or extremely severe) according to established cutoff values (see Appendices for detailed cutoff criteria).

Statistical analysis

Data were analyzed using IBM SPSS Statistics software (IBM Corp., Armonk, NY). Descriptive statistics (means, standard deviations (SD), and percentages) were calculated. Independent samples t-tests were used to compare continuous variables between groups. Chi-square tests were used to compare categorical variables and examine the overall distribution of mental health symptom patterns between groups. Statistical significance was set at p≤0.05.

## Results

A total of 190 adolescents participated in this cross-sectional study, with 95 participants in each group (Hatha yoga and recreational sports). The sample comprised 101 male participants (53.16%) and 89 female participants (46.84%), with ages ranging from 10 to 19 years according to WHO age criteria.

Significant demographic differences were observed between groups (Table 1). The Hatha yoga group was predominantly older adolescents (56.84% aged 15-19 years) with female predominance (53.68%), while the recreational sports group consisted primarily of younger adolescents (77.89% aged 10-14 years) with male predominance (67.37%) (χ²=23.99, p<0.001 and χ²=8.582, p=0.003, respectively). Sleep patterns showed no significant differences between groups (χ²=4.462, p=0.181), with most participants in both groups reporting 6-8 hours of sleep nightly. Social media use was also comparable between groups (χ²=1.131, p=0.563).

**Table 1:** Demographic and behavioral characteristics of adolescents by physical activity type Demographic and behavioral characteristics by physical activity type. Data shown as number (%). Chi-square tests used for group comparisons; p<0.05 considered statistically significant.

Variable category	Hatha yoga (n=95)	Recreational sports (n=95)	Chi-square	p-value
Age group			23.99	<0.001
10-14 years	41 (43.16%)	74 (77.89%)		
15-19 years	54 (56.84%)	21 (22.11%)		
Gender			8.582	0.003
Male	44 (46.32%)	64 (67.37%)		
Female	51 (53.68%)	31 (32.63%)		
Hours of sleep			4.462	0.181
Less than 6 hours	0 (0%)	3 (3.16%)		
6-8 hours	66 (69.47%)	71 (74.74%)		
More than 8 hours	29 (30.53%)	21 (22.11%)		
Social media use			1.131	0.563
No use	30 (31.58%)	32 (33.68%)		
0-2 hours	56 (58.95%)	50 (52.63%)		
More than 2 hours	9 (9.47%)	13 (13.68%)		
Hours of practice			95.786	<0.001
1-2 hours	79 (83.16%)	14 (14.74%)		
2-7 hours	16 (16.84%)	43 (45.26%)		
More than 7 hours	0 (0%)	38 (40%)		

The groups demonstrated significant differences in age and practice intensity patterns (Table 2). The yoga group was significantly older than the sports group (14.51±1.63 versus 13.14±2.4 years, p<0.001). Most notably, the sports group engaged in significantly more weekly practice hours than the yoga group (7.77±5.26 versus 3.14±2.4 hours, p<0.001), representing a 2.5-fold difference in activity exposure. Sleep duration (7.97±1.06 versus 7.91±1.36 hours, p=0.386) and social media use (1.28±1.31 versus 1.07±1.8 hours, p=0.362) showed no significant differences between groups.

**Table 2:** Comparison of age, sleep, social media use, practice hours, and mental health scores between Hatha yoga and recreational sports groups Data are presented as mean±SD. Independent samples t-tests were conducted to compare group means, with degrees of freedom = 188. Significance level set at p<0.05. SD: standard deviation

Parameter	Hatha yoga (mean±SD)	Sport group (mean±SD)	t(188)	p-value
Age (years)	14.51±1.63	13.14±2.4	4.603	<0.001
Sleep (hours)	7.97±1.06	7.91±1.36	0.339	0.386
Social media use (hours)	1.28±1.31	1.07±1.8	0.919	0.362
Hours of practice	3.14±2.4	7.77±5.26	-7.805	<0.001
Stress scores	13.7±6.18	11.75±7.44	1.965	0.05
Anxiety scores	10.55±7.08	11.33±8.46	-0.689	0.447
Depression scores	10.46±6.28	9.05±7.84	1.368	0.149

Mental health assessment using DASS-21 subscales revealed one significant difference between groups (Table 2). The recreational sports group demonstrated significantly lower stress scores compared to the Hatha yoga group (11.75±7.44 versus 13.70±6.18, p=0.050). No significant differences were observed for anxiety scores (11.33±8.46 versus 10.55±7.08, p=0.447) or depression scores (9.05±7.84 versus 10.46±6.28, p=0.149).

**Figure 1:**
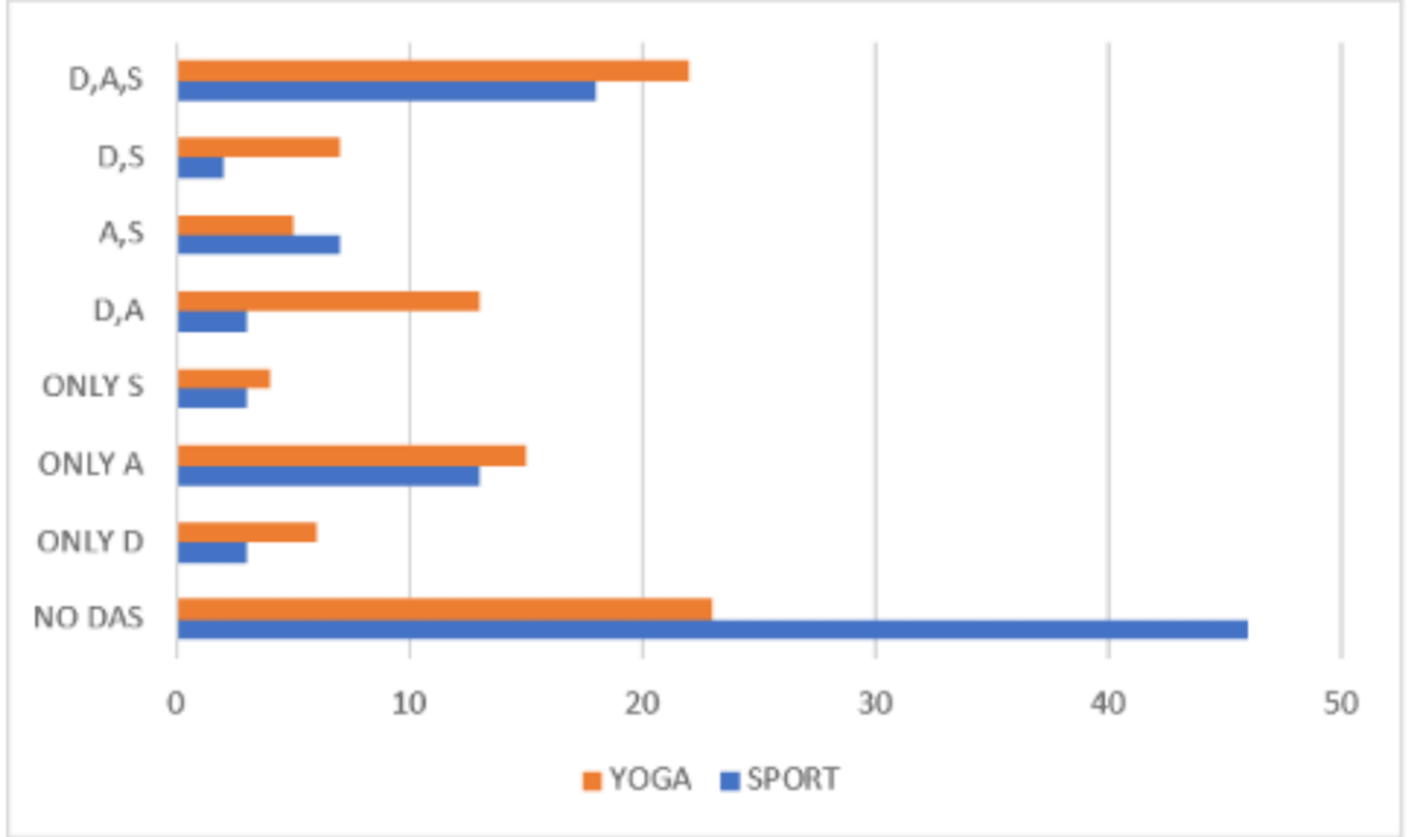
Mental status according to the DASS in the Hatha yoga group and recreational sports group The horizontal bar chart shows the frequency of participants experiencing different combinations of D, A, and S symptoms in Hatha yoga (orange bars) and recreational sports (blue bars) groups. NO DAS: no depression, anxiety, or stress symptoms, D,A,S: all three conditions present, individual letters represent single conditions, combinations represent co-occurring symptoms Total participants per group: yoga: n=95, sports: n=95 DASS: Depression Anxiety Stress Scales, D: depression, A: anxiety, S: stress

Chi-square analysis revealed significant overall differences in mental health symptom distribution patterns between groups (χ²=17.618, df=3, p<0.001) (Table 3). The recreational sports group demonstrated a notably higher proportion of asymptomatic participants compared to the yoga group (48.4% versus 22.1%). Conversely, the yoga group showed higher rates of participants experiencing two co-occurring conditions (32.6% versus 13.7%). Single condition presentations were similar between groups (20% versus 21.1%), as were rates of all three conditions combined (17.9% versus 24.2%) (Figure 1).

**Table 3:** Distribution of mental health symptom patterns between Hatha yoga and recreational sports groups Data presented as number (%). Chi-square tests used for group comparisons. Single condition: depression only, anxiety only, or stress only, two conditions: any combination of two symptoms, all three conditions: depression, anxiety, and stress present simultaneously Chi-square statistic: 17.618, degrees of freedom: 3, p-value: <0.001

Symptom category	Hatha yoga (n=95)	Hatha yoga (%)	Recreational sports (n=95)	Recreational sports (%)
No symptoms	21	22.10%	46	48.40%
Single condition	20	21.10%	19	20%
Two conditions	31	32.60%	13	13.70%
All three conditions	23	24.20%	17	17.90%
Total	95	100%	95	100%

## Discussion

This cross-sectional study revealed significant differences in overall mental health outcomes between adolescents engaged in recreational sports compared to those engaged in Hatha yoga practice, with recreational sports participants demonstrating superior mental health profiles across multiple domains. Specifically, recreational sports participants showed significantly lower stress scores (11.75±7.44 versus 13.70±6.18) and better overall mental health outcomes compared to yoga practitioners. However, substantial confounding factors, including notable differences in practice duration, age distribution, and potential self-selection bias, necessitate cautious interpretation of these results as descriptive rather than causal evidence.

The observed differences in stress levels may be associated with distinct neurobiological pathways. Yoga primarily downregulates the hypothalamic-pituitary-adrenal axis and sympathetic nervous system, decreasing cortisol and catecholamine levels while enhancing GABA and serotonin signaling [23-25]. Conversely, recreational sports are associated with an increase in monoamine neurotransmitters and promote brain-derived neurotrophic factor release, mechanisms resembling selective serotonin reuptake inhibitor effects [26]. Recreational sports, whether involving aerobic components or anaerobic intervals like sprinting phases in basketball and football, generate acute neurochemical responses, including endorphin release and improved mood regulation. The intermittent high-intensity nature of many recreational sports combines both aerobic and anaerobic elements, potentially maximizing neurobiological benefits through varied physiological stress and recovery cycles. In contrast, Hatha yoga's low-intensity practice (1-2 METs) primarily activates parasympathetic pathways, elevating brain GABA levels and downregulating the hypothalamic-pituitary-adrenal axis and sympathetic nervous system activity [11]. The social dynamics inherent in team-based recreational activities may provide additional stress-buffering effects through peer support and collective efficacy, contrasting with the introspective nature of individual yoga practice [27].

These mechanistic differences are supported by recent research. Pasquerella et al. demonstrated that strategic team sports significantly reduce adolescent stress through enhanced social dynamics, while individual sports primarily improve cognitive flexibility [28]. Similarly, Sharma et al. found that yoga effects manifest immediately, whereas exercise benefits develop over time, suggesting temporally distinct adaptation processes [29]. This temporal divergence may partly explain our cross-sectional findings, as participants had been engaged in their respective activities for varying durations.

The demographic distribution provides a crucial interpretive context that may be a confounding factor. Sports participants were younger (13.14 versus 14.51 years) and practiced substantially more hours weekly (7.77 versus 3.14 hours). This age difference aligns with developmental patterns showing increased stress during mid-to-late adolescence due to academic pressures and identity formation challenges [30]. The demographic differences between groups represent a significant confounding factor, as gender disparities in adolescent depression rates are well-established [31], suggesting that our findings may reflect underlying gender-based vulnerabilities rather than activity-specific effects. The 2.5-fold practice duration disparity suggests a dose-response relationship, consistent with systematic reviews demonstrating enhanced mental health benefits with greater physical activity exposure frequency and duration [32,33]. Therefore, the observed stress differences may reflect age-related stress patterns and activity exposure rather than group-specific effects.

The symptom distribution pattern analysis revealed the most clinically significant finding of this study. Recreational sports participants were more than twice as likely to report no detectable mental health symptoms compared to yoga practitioners (48.4% versus 22.1%), representing a substantial population-level difference. This finding extends beyond the observed stress score differences and suggests that the association between recreational sports participation and mental health may be more comprehensive than individual symptom measurements indicate. Conversely, yoga participants demonstrated higher rates of experiencing two co-occurring conditions (32.6% versus 13.7%), suggesting a tendency toward multiple symptom presentations rather than isolated conditions. These patterns may reflect different mental health trajectories, with sports participants clustering toward either complete wellness or more severe presentations, while yoga participants show intermediate symptom complexity. However, this cross-sectional design cannot determine whether these patterns reflect the influence of physical activities on mental health development, differential help-seeking behaviors, or pre-existing mental health differences that influence activity selection.

The significant difference in practice intensity between groups (7.77 versus 3.14 hours weekly) represents both a major confounding factor and a meaningful finding that provides important evidence supporting a dose-response relationship between physical activity volume and mental health benefits [32,33]. While this dose difference limits our ability to attribute stress reduction specifically to sports versus yoga, the sports group's greater practice hours may partially or entirely explain their superior mental health outcomes, suggesting that the quantity of movement-based activity may be as important as, or more important than, the specific type of activity for adolescent mental health. This finding aligns with established literature demonstrating dose-response relationships in exercise interventions and suggests that adolescent mental health programs should prioritize achieving higher volumes of physical activity engagement, regardless of the specific modality chosen. The sports group's naturally higher engagement levels (averaging nearly eight hours weekly) may reflect the inherently motivating and social nature of team-based activities, suggesting that sports programs may offer advantages in sustaining long-term participation compared to individual practices, which has important implications for program design and implementation.

This study possesses several notable strengths. It provides novel comparative data between distinct physical activity modalities: mind-body practice versus recreational sports during adolescence, a critical period when mental health conditions typically emerge. The validated DASS-21 instrument enabled assessment of three psychological dimensions, while the adequate sample size (N=190) based on formal power calculations addresses a common limitation in yoga research. Importantly, beyond comparing mean scores, this study examined symptom distribution and co-occurrence patterns, revealing that 48% of sports participants reported no symptoms compared to 22% of yoga practitioners, a clinically meaningful difference that aggregate statistics would obscure. The systematic assessment of confounding variables, transparent acknowledgment of limitations, and real-world community-based design enhance methodological rigor and ecological validity. Finally, by examining accessible, school-implementable interventions, this research addresses practical considerations for population-level mental health promotion.

A critical unmeasured confounding factor is the potential for baseline mental health differences between groups. Individuals may self-select into different physical activities based on their existing mental health status, personality traits, or coping preferences. Adolescents with higher baseline stress might gravitate toward introspective practices such as yoga, while those with better mental health might prefer social, dynamic activities such as recreational sports [34]. This selection bias could entirely explain our observed differences, as we cannot establish the temporal relationship between activity participation and mental health status. Additionally, the study did not control for socioeconomic status, family support, or concurrent mental health interventions, all of which could influence outcomes [35]. While all participants attended private schools in urban Bengaluru, providing some socioeconomic homogeneity, unmeasured variability in family income, parental education, and social support systems may have influenced findings. However, observational studies can still provide valuable insights when their limitations are clearly acknowledged [36].

Despite these limitations, the findings have important clinical and public health implications [37]. Recreational sports programs offer advantages over traditional mental health interventions, including cost-effectiveness, scalability through existing educational infrastructure, and accessibility without specialized training [38]. Implementation could leverage physical education curricula and community partnerships to address mental health provider shortages during the ongoing adolescent mental health crisis. Future research requires randomized controlled trials with matched populations, standardized protocols, and equivalent exposure duration to establish comparative effectiveness. These findings suggest potential associations between recreational sports and adolescent mental health through distinct neurobiological and social mechanisms, but methodological limitations necessitate cautious interpretation and validation through controlled studies before definitive clinical recommendations [39,40].

## Conclusions

This cross-sectional study revealed significantly better overall mental health outcomes, including lower stress scores and a higher proportion of asymptomatic participants, among adolescents engaged in recreational sports compared to Hatha yoga practice. However, this study provides descriptive evidence of differing mental health patterns rather than establishing causal relationships between these activities and mental health outcomes. The observed differences may reflect distinct demographic characteristics and self-selection patterns, with sports attracting younger male participants practicing significantly more hours weekly and yoga drawing older female participants.

While these findings cannot establish causal relationships due to the cross-sectional design and potential confounding factors, they provide valuable preliminary evidence supporting the incorporation of accessible physical activities into school-based mental health initiatives. The substantial between-group differences observed, particularly in symptom-free rates, warrant further investigation through randomized controlled trials with matched populations and standardized intervention protocols. Given that the majority of adolescent mental health conditions remain unrecognized and untreated, these findings generate important hypotheses regarding the potential differential effects of various physical activity modalities on adolescent mental health, but controlled studies are essential to establish definitive causal relationships and guide evidence-based policy recommendations for adolescent mental health promotion.

## Figures and Tables

**Figure 2:**
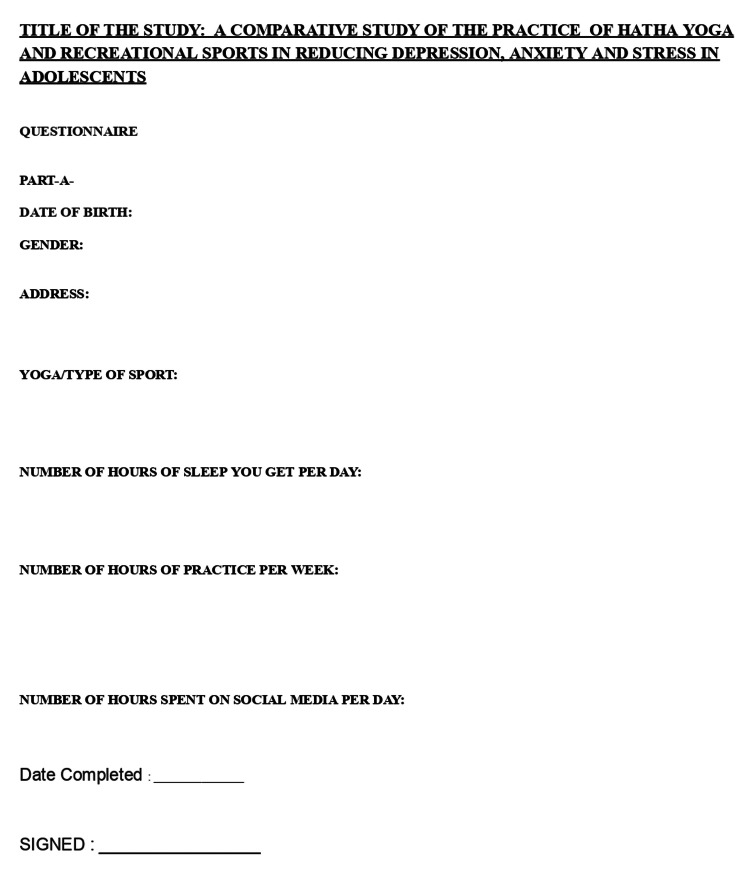
Page 1 of the questionnaire administered to the participants

**Figure 3:**
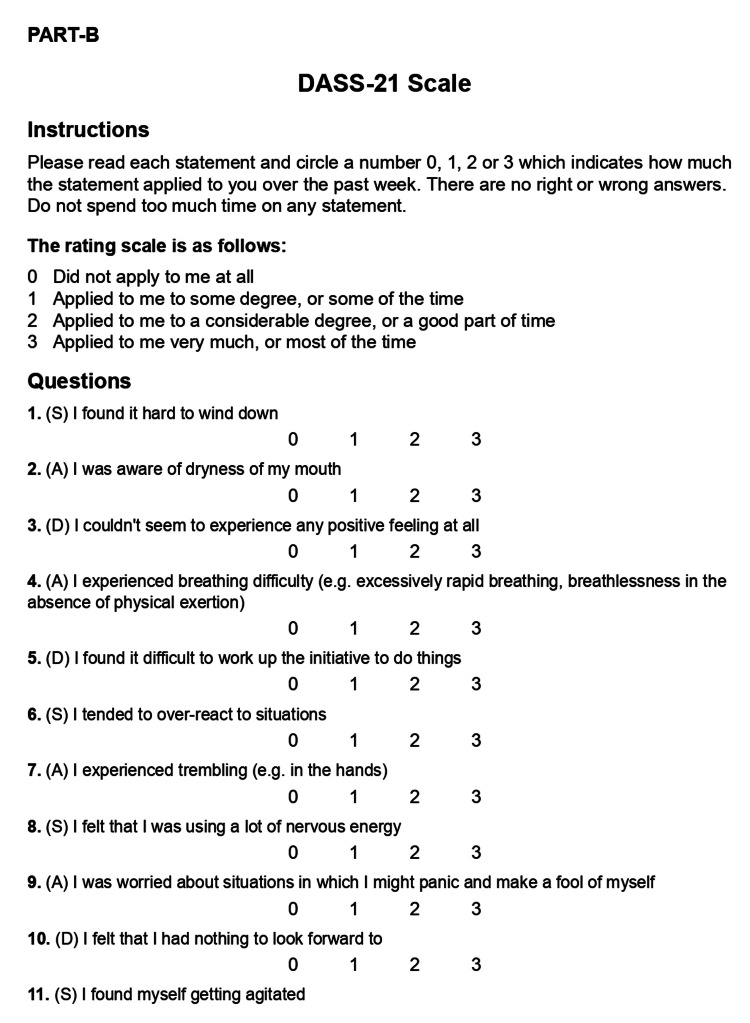
Page 2 of the questionnaire administered to the participants DASS-21: Depression Anxiety Stress Scales, D: depression, A: anxiety, S: stress

**Figure 4:**
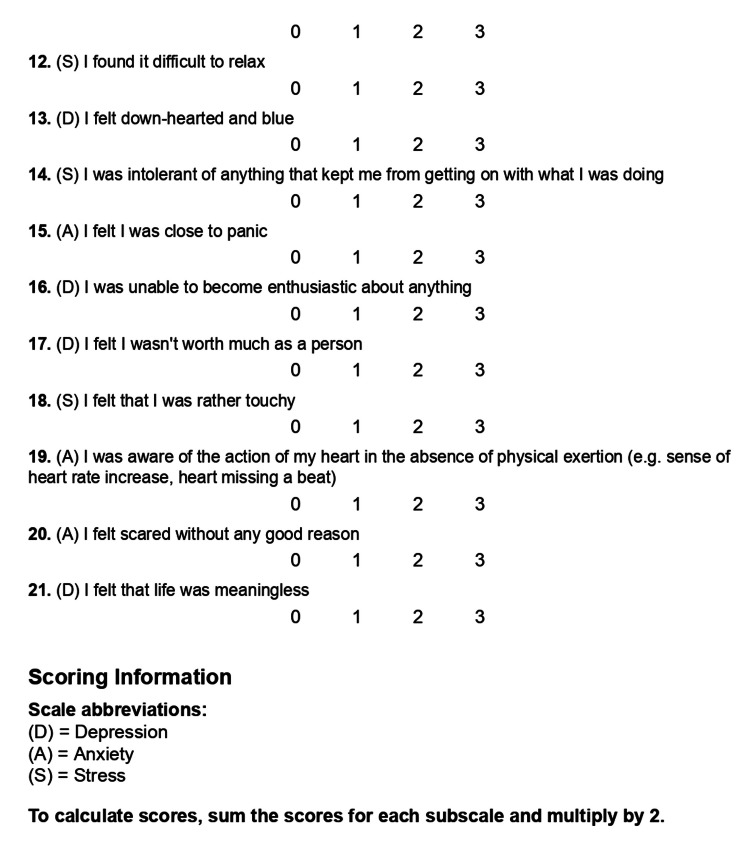
Page 3 of the questionnaire administered to the participants D: depression, A: anxiety, S: stress

## Appendices

The questionnaire used in this study is shown in Figures 2-4. 
